# Development and Validation of a New LC-MS/MS Bioanalytical Method for the Simultaneous Determination of Levodopa, Levodopa Methyl Ester, and Carbidopa in Human Plasma Samples

**DOI:** 10.3390/molecules28114264

**Published:** 2023-05-23

**Authors:** Linda Molteni, Bruno Charlier, Viviana Izzo, Albino Coglianese, Valeria Conti, Roberto Eleopra, Roberto Cilia, Chiara Capelli, Annachiara D’Urso, Ugo de Grazia

**Affiliations:** 1 Laboratory of Neurological Biochemistry and Neuropharmacology, Fondazione IRCCS Istituto Neurologico Carlo Besta, 20133 Milano, Italy; chiara.capelli02@universitadipavia.it (C.C.); annachiara.durso@istituto-besta.it (A.D.); ugo.degrazia@istituto-besta.it (U.d.G.); 2 Department of Medicine, Surgery and Dentistry “Scuola Medica Salernitana”, University of Salerno, Baronissi, 84081 Salerno, Italy; bcharlier@unisa.it (B.C.); vizzo@unisa.it (V.I.); albino.cog@gmail.com (A.C.); vconti@unisa.it (V.C.); 3 University Hospital “San Giovanni di Dio e Ruggi d’Aragona”, 84131 Salerno, Italy; 4 Graduate School in Clinical Pathology and Clinical Biochemistry, University of Salerno, Baronissi, 84081 Salerno, Italy; 5 Parkinson and Movement Disorders Unit, Department of Clinical Neurosciences, Fondazione IRCCS Istituto Neurologico Carlo Besta, 20133 Milano, Italy; roberto.eleopra@istituto-besta.it

**Keywords:** levodopa, levodopa methyl ester, carbidopa, Parkinson’s disease, therapeutic drug monitoring, liquid chromatography–mass spectrometry (LC–MS), analytical method

## Abstract

Levodopa (L-DOPA) treatment, combined with the administration of dopa-decarboxylase inhibitors (DDCIs), is still the most effective symptomatic treatment of Parkinson’s disease (PD). Although its efficacy in the early stage of the disease has been confirmed, its complex pharmacokinetics (PK) increases the variability of the intra-individual motor response, thus amplifying the risk of motor/non-motor fluctuations and dyskinesia. Moreover, it has been demonstrated that L-DOPA PK is strongly influenced by several clinical, therapeutic, and lifestyle variables (e.g., dietary proteins). L-DOPA therapeutic monitoring is therefore crucial to provide personalized therapy, hence improving drug efficacy and safety. To this aim, we have developed and validated an ultra-high performance liquid chromatography–tandem mass spectrometry (UHPLC-MS/MS) method to quantify L-DOPA, levodopa methyl ester (LDME), and the DDCI carbidopa in human plasma. The compounds were extracted by protein precipitation and samples were analyzed with a triple quadrupole mass spectrometer. The method showed good selectivity and specificity for all compounds. No carryover was observed, and dilution integrity was demonstrated. No matrix effect could be retrieved; intra-day and inter-day precision and accuracy values met the acceptance criteria. Reinjection reproducibility was assessed. The described method was successfully applied to a 45-year-old male patient to compare the pharmacokinetic behavior of an L-DOPA-based medical treatment involving commercially available *Mucuna pruriens* extracts and an LDME/carbidopa (100/25 mg) formulation.

## 1. Introduction

Parkinson’s disease (PD) is a common neurodegenerative condition characterized by the progressive loss of dopaminergic neurons and subsequent alteration in dopaminergic transmission at nigrostriatal level [1,2].

Dopaminergic substitution is considered the main therapeutic strategy for the symptomatic treatment of PD [3,4]. The administration of the dopamine precursor levodopa (L-DOPA) has remained the main cardinal treatment since its introduction in the 1960s [3,5,6]. Due to its limited bioavailability and the frequent manifestation of adverse events [7], L-DOPA has been promptly combined with the administration of peripheral dopa-decarboxylase inhibitors (DDCIs), which proved to be able to hinder L-amino acid decarboxylase (AADC) activity in the peripheral compartment (mainly at gastrointestinal level) [8,9]. Incapable of crossing the blood-brain barrier (BBB), DDCIs, such as carbidopa and benserazide, increase the concentration of dopamine (DA) in the brain by preventing the bio-activation of L-DOPA at the peripheral level, thus potentiating its therapeutic effect [4,8].

While the introduction of DDCIs has increased the central bioavailability and therapeutic action of L-DOPA by reducing its peripheral metabolism rate, the progression of nigrostriatal terminals loss and individual daily doses of long-term treatment with L-DOPA/DDCIs increase the risk of developing motor/non-motor fluctuations and dyskinesia [10,11,12]. The occurrence of motor complications is due to several factors, including progressive loss of presynaptic dopaminergic regulation (e.g., dopamine transporter, pre-synaptic D2 receptors), hindering of the buffering ability against alterations in L-DOPA levels, irregular gastrointestinal emptying, delayed L-DOPA absorption and L-DOPA short plasma half-life [10,11,12,13]. Thus, a strict correlation between L-DOPA-related motor complications and its complex pharmacokinetics (PK) can be evinced. In addition, L-DOPA PK can be strongly influenced by several demographic, clinical, and lifestyle factors, such as gender, body mass index (BMI), age, disease duration, and diet (protein redistribution dietary regimen is in fact recommended) [14,15,16,17,18,19,20,21]. Among those, gender seems to be a strong predictor of L-DOPA PK: women show greater L-DOPA bioavailability, higher plasma concentrations, as well as higher AUC and Cmax, and are also more prone to develop L-DOPA-related complications when compared to men [14,15].

In the past decades, the association between motor fluctuations and L-DOPA PK mechanisms became clearer and various strategies were explored to increase L-DOPA bioavailability, particularly by enhancing its absorption. In this context, a more soluble L-DOPA prodrug, levodopa methyl ester (LDME), was introduced [22]. Considering that L-DOPA is still largely unavailable and unaffordable for the majority of patients in many Sub-Saharan African and other low-income countries, growing interest has been also directed towards a leguminous plant, *Mucuna pruriens* variant *utilis*, which seeds are reported to be rich in L-DOPA. Recent studies have shown that *Mucuna pruriens* powder is able to control motor and non-motor symptoms of PD, with a different PK compared to marketed L-DOPA formulations. *Mucuna pruriens* seeds may represent an effective alternative to L-DOPA conventional treatment in low-income areas, where it is unaffordable and inaccessible, although tolerability in the long term is still under investigation [23,24,25,26].

In this complex scenario, it is of crucial importance to monitor L-DOPA plasma concentrations to integrate PK data with clinical and demographic information to identify additional predictors of L-DOPA PK within lifestyle and therapeutic variables, which could be really useful to optimize and personalize dopaminergic therapy, thus ensuring drug efficacy and safety [14,15]. Recently, Conti and coworkers [14] described the use of a novel LC-MS/MS method to evaluate L-DOPA plasma concentrations in a clinical study, which aim was to identify L-DOPA PK predictors. In this work, 35 L-DOPA-naïve patients, 19 men and 16 women, were enrolled in a multi-center study involving different Italian hospitals and were treated with a single dose of oral L-DOPA/benserazide (100/25 mg) formulation. Multiple regression analyses were applied to identify PK predictors amongst variables such as gender, age, and BMI; the female sex turned out as a strong predictor of L-DOPA AUC and Cmax, while BMI was identified as a predictor of AUC only. Moreover, only BMI was shown to predict t_1/2_; when stratifying by gender, BMI was confirmed to significantly predict t_1/2_ only in women. According to the results described in this study, women are more prone to develop motor complications when compared to men and this is reflected in higher plasma concentrations, as well as higher Cmax and AUC. Although useful for its purpose, the main limitation of this methodology was the ability to evaluate the sole L-DOPA plasma concentration. Various LC-MS/MS methods were developed to monitor L-DOPA and carbidopa in human plasma [27,28,29,30,31,32,33], but to date, no method was found in the scientific literature for the monitoring of LDME in LC-MS/MS.

In this work, we have developed and validated, according to the most recent guidelines on bioanalytical method validation and study sample analysis [34], a UHPLC-MS/MS method for the simultaneous determination of L-DOPA, LDME, and carbidopa in human plasma. The novel bioanalytical method relies on a simple acidic protein precipitation extraction procedure and provides a rapid simultaneous determination of the pharmacologically active compound L-DOPA, as well as its prodrug LDME and one of the two known DDCIs, carbidopa. The development of this method has allowed performing a study on a 45-year-old male patient aimed at the comparison of the pharmacokinetic behavior of two different L-DOPA-containing formulations, i.e., commercially available *Mucuna pruriens* extracts and LDME/carbidopa (100/25 mg) tablets.

## 2. Results

### 2.1. Method Validation

Method validation was performed according to the most recent ICH guidelines for bioanalytical method validation [34], which require the following assays: selectivity and specificity, calibration curve linearity, carry-over, dilution integrity, matrix effect, accuracy and precision, reinjection reproducibility, and stability. Technical details are explained in Section 4.5.

Method selectivity and specificity were assessed by analyzing blank plasma samples from different matrices; no interfering peaks with response higher than 20% and 5%, when compared to lower limit of quantification (LLOQ) and internal standard (IS) signals, were found for any of the compounds tested. 

**Figure 1:**
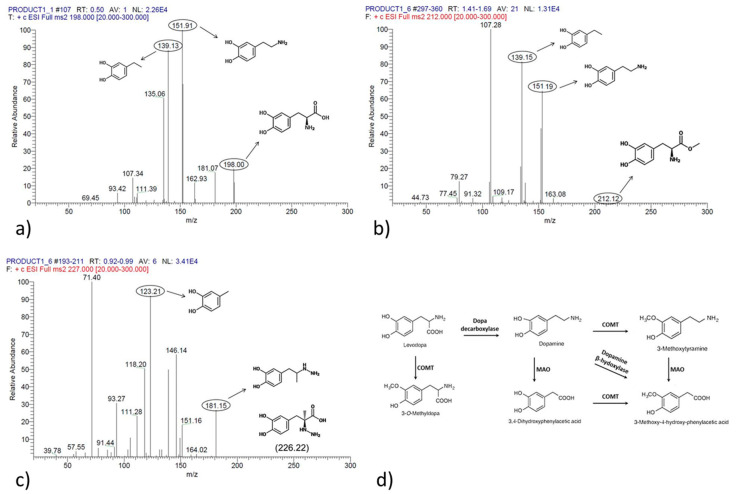
Spectral data and fragment analysis of L-DOPA (panel (**a**)), LDME (panel (**b**)), and carbidopa (panel (**c**)). Panel (**d**) shows L-DOPA metabolism and enzymes involved in sequential transformation (catechol-O-methyltransferase COMT; monoamine oxidases (MAO).

**Figure 2:**
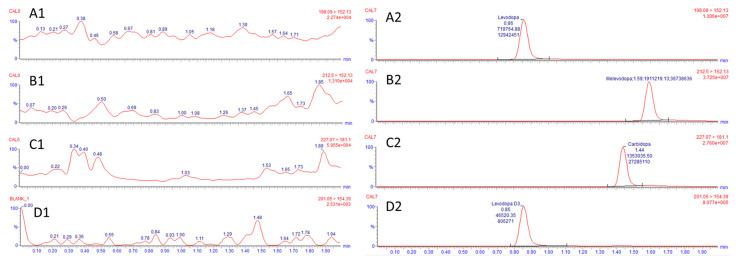
Representative chromatograms of blank plasma (**1**) compared to spiked plasma (**2**). In panel (**A**), L-DOPA (quan 198.09 → 152.13 *m/z*, RT: 0.89 min); in panel (**B**), LDME (quan 212.50 → 152.13 *m/z*, RT: 1.53 min); in panel (**C**), carbidopa (quan 227.07 → 181.11 *m/z*, RT: 1.44 min); in panel (**D**), internal standard L-DOPA-D3 (201.05 → 154.39 *m/z*, RT: 0.85 min). Qualifier ions are described in Table 3.

Figure 1 shows spectral data and fragmentation analysis of L-DOPA, LDME, and carbidopa, and Figure 2 shows representative chromatograms of blank plasma and samples spiked with L-DOPA, LDME, and carbidopa.

Our method showed to be linear up to 10,000 µg/L L-DOPA and LDME and 6000 µg/L for carbidopa (ULOQ); LLOQ was set at 15 µg/L for all analytes. Although the method is linear up to higher concentration levels, the calibration curve upper quantification limit was set at 2000 µg/L for clinical purposes.

A 1/x weighted linear regression model was applied and the method showed to be linear over the calibration range and beyond. Representative linear equations and regression coefficients are: for L-DOPA, y = 0.00785897x + 0.029, R^2^ = 0.998; for LDME, y = 0.0246945x − 0.117, R^2^ = 0.995; for carbidopa, y = 0.015161x + 0.004, R^2^ = 0.998.

Negligible carryover was observed: six blank injections after samples at ULOQ showed a mean signal of 6.8 ± 1.9% of LLOQ response for L-DOPA, 5.6 ± 6.0% of LLOQ response for LDME, 3.1 ± 2.5% of LLOQ response for carbidopa, and 0.028 ± 0.014% of IS signal for deuterated levodopa (L-DOPA-D3).

Dilution integrity was assessed by measuring the precision and accuracy of diluted samples (*n* = 5): mean bias% and CV% were, respectively, 1.2% and 1.8% for L-DOPA, 2.7% and 1.9% for LDME, and 3.9% and 2.1% for carbidopa.

No matrix effect (ME) could be found for any of the compounds when measured at low-quality control level (LQC), medium QC level (MQC), and high QC level (HQC) concentrations (as shown in Supplementary Material, Table S1). Mean IS-normalized ME (ISn-ME) values were 106 ± 3.2% for L-DOPA, 98 ± 6.2% for LDME, 107 ± 2.5% for carbidopa, and 97 ± 3.6% for L-DOPA-D3. Mean IS-normalized recovery (ISn-RE) of plasma extraction was 110 ± 3.5% for L-DOPA, 79 ± 3.8% for LDME, 93 ± 3.2% for carbidopa, and 98 ± 5.6% for L-DOPA-D3 (Table S1).

Precision and accuracy were assessed at LLOQ, LQC, MQC, and HQC following a 5 × 5 scheme. Bias% and CV% values for inter-day and intra-day measurements are shown in Table 1a,b, respectively. All values met the acceptance criteria.

Reinjection reproducibility was evaluated by measuring the accuracy and precision of a reinjected run. All values were within the acceptance criteria.

Due to the well-known high instability of catecholamines and affiliated compounds, short-term and medium-term stability was assessed (as shown in Supplementary Materials, Figure S1). Short-term stability at room temperature under both light exposure and light protection conditions and at 4 °C was unsatisfactory for all compounds, with the exception of carbidopa when stored at 4 °C. On the other hand, extracts stored in the autosampler compartment were stable for up to 1 week for all compounds. Most importantly, L-DOPA and carbidopa showed good stability in plasma samples when stored at −40 °C up to 2 months and 1 month, respectively; in the same condition, LDME stability was not satisfactory.

### 2.2. Pharmacokinetic Study

**Figure 3:**
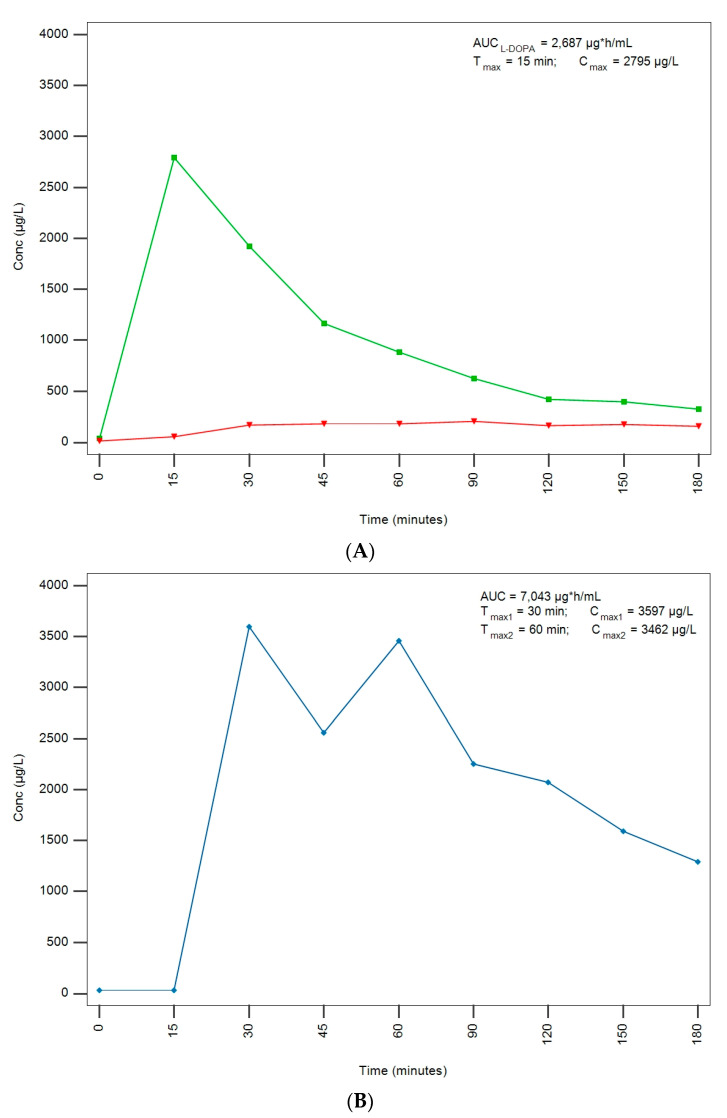
L-DOPA plasma concentrations (µg/L) and PK parameters at 0, 15, 30, 45, 60, 90, 120, 150, and 180 min after administration of two tablets of an LDME/carbidopa (100/25 mg) formulation (L-DOPA concentrations curve in green and carbidopa in red) (**A**) compared to plasma concentrations after the administration of 500 mg of L-DOPA from commercially available *Mucuna pruriens* extracts (*Mucuna pruriens*-derived L-DOPA concentrations curve in blue) (**B**).

The developed and validated method was applied to real samples obtained from a male patient under treatment at Fondazione IRCCS Istituto Neurologico Carlo Besta. A 45-year-old male patient who was reported to medicate with commercially available *Mucuna pruriens* extracts alone was examined. L-DOPA PK parameters after administration of two tablets of an LDME/carbidopa (100/25 mg) formulation were compared with those obtained after the administration of 500 mg of L-DOPA from *Mucuna pruriens* extracts in off-med conditions from 12 h before (previously treated with L-DOPA/benserazide). In order to maintain the same L-DOPA central bioavailability within the treatments, the *Mucuna pruriens* dose was adjusted by multiplying the conventional L-DOPA dose by five times, hence simulating the theoric absence of DDCIs in *Mucuna pruriens* extract [24]. L-DOPA PK parameters are summarized in Table 2 and plasma concentrations are plotted in Figure 3.

LDME and carbidopa plasma concentrations were also monitored. No LDME could be found when the patient was treated with LDME/carbidopa formulation, thus confirming its complete degradation as a prodrug; carbidopa was detected and its concentration curve, along with L-DOPA, is also shown in Figure 3. No LDME and negligible carbidopa concentrations were detected when the patient was treated with *Mucuna pruriens* extracts.

Noteworthily, the treatment with *Mucuna pruriens* extracts determined higher Cmax and higher AUC compared to the treatment with conventional LDME/carbidopa formulation, in line with the results obtained by Cilia et al. [24]. On the other hand, Tmax was lower when the patient was treated with LDME/carbidopa formulation (Figure 3). The administration of *Mucuna pruriens* extracts was also characterized by higher Cmin and t_1/2_ values when compared with conventional L-DOPA treatment, hence indicating a slower elimination rate. 

PK parameters measured after the administration of *Mucuna pruriens* extracts were compared with those presented by Cilia et al. [24]: plasmatic concentration curves in patients administered *Mucuna pruriens* extracts often show double maximum L-DOPA concentration peaks, thus confirming irregular *Mucuna pruriens* PK. It can also be evinced that the activity of the DDCI benserazide was depleted during the 12 h in off-med condition, otherwise, L-DOPA plasma concentration would have been 5 times higher due to inhibition of gastrointestinal AADC activity, which is not hindered when only *Mucuna pruriens* extracts are administered.

## 3. Discussion

L-DOPA/DDCIs administration is considered the mainstay therapeutic strategy for PD treatment, and its efficacy in the early stage of the disease has been extensively reported [2,3,4,5]; with the protracting of the disease, the manifestation of motor complications associated with L-DOPA administration becomes more frequent and causes a rapid decrease in PD patients’ quality of life [10,11,12,13]. As the occurrence of motor complications is strictly associated with the complex L-DOPA PK, the therapeutic monitoring of L-DOPA plasma concentrations plays a crucial role in the management of PD patients [14,15]. In fact, L-DOPA PK is influenced by several clinical, therapeutic, and lifestyle variables, such as gender, BMI, age, disease duration, and severity [14,15,16,17,18,19,20,21]. The identification of other predictors of L-DOPA PK and the integration of these data with plasma concentrations and clinical information could be really helpful to assist clinicians in the optimization of personalized PD therapy, hence ensuring drug efficacy and safety [14,15].

To this aim, we have successfully developed and validated a novel UHPLC-MS/MS bioanalytical method able to quantify in a single run L-DOPA, LDME, and carbidopa in human plasma samples. This method provides the quantification of the pharmacologically active compound, and most importantly allows the monitoring of plasma concentrations of the prodrug LDME and of one of the two known DDCIs, carbidopa, thus ensuring a comprehensive understanding of L-DOPA PK and its alterations. Unfortunately, we could not manage to include the DDCI benserazide due to severe stability issues of the compound; a future perspective will be the overcoming of these issues and the introduction of benserazide within the developed method. 

The method proved to be sensitive and selective towards the compounds and was successfully validated according to the most recent guidelines for bioanalytical method validation [34]. Carryover, dilution integrity, matrix effect, accuracy and precision, reinjection reproducibility, and stability were investigated. All parameters were within the acceptance criteria. Nevertheless, the main difficulty we encountered during method validation was the low stability of the compounds in the matrix, in particular when stored at room temperature or at 4 °C. Moreover, LDME was not stable in any of the conditions tested while the other compounds were stable only when frozen and stored at −40 °C up to 1 month for carbidopa and to 2 months for L-DOPA. For this reason, study samples should be processed shortly after drawing to ensure accurate determination of all compounds. Samples should be always handled at low temperatures (e.g., ice bucket, refrigerated centrifuge); samples could also be stored at −40 °C but this process may alter LDME plasma concentrations. Overall, the method performance was good, providing the simultaneous determination of the compound with a short run time and a simple extraction procedure.

As a proof of concept, our method was applied to measure PK parameters in a patient treated with commercially available *Mucuna pruriens* extracts. L-DOPA PK parameters after administration of two tablets of an LDME/carbidopa (100/25 mg) formulation were compared with those obtained after the administration of 500 mg of L-DOPA from *Mucuna pruriens* extracts in off-med conditions from 12 h before. LDME and carbidopa plasma concentrations were also monitored.

The application to real-life PD patients’ samples showed that our validated method could be useful to simultaneously monitor L-DOPA plasma concentrations, as well as those of the prodrug LDME and of the DDCI carbidopa, providing a complete overview of L-DOPA PK and its eventual alterations. Indeed, the therapeutic monitoring of L-DOPA and the integration of PK data with clinical and demographic information could be valuable tools for the optimization of what is considered the “gold standard” in the symptomatic treatment of PD.

## 4. Materials and Methods

### 4.1. Chemicals and Reagents

L-DOPA, LDME, carbidopa, and L-DOPA-D3 standards were purchased by Merck (Milan, Italy). Stock solutions were prepared in 10 mM HCl at the nominal concentration of 150 µg/mL for L-DOPA, LDME, carbidopa and at the nominal concentration of 50 µg/mL for L-DOPA-D3. Stock solutions were stored at −20 °C.

Trifluoroacetic acid (TFA) was purchased by Merck (Milan, Italy). Ultrapure water was obtained from a Milli-Q water purification system (Millipore, Milan, Italy). LC-MS grade acetonitrile and formic acid were purchased by Merck (Milan, Italy).

### 4.2. Calibration Curves and Quality Control Samples

Blank plasma from healthy donors was spiked to obtain an eight-point calibration curve (0, 125, 250, 500, 750, 1000, 1500, 2000 µg/L), as well as 3 levels of QC samples at low (LQC, 300 µg/L), medium (MQC, 900 µg/L) and high (HQC, 1800 µg/L) concentrations.

Freshly spiked calibration curves and quality control samples were prepared before each analytical run.

### 4.3. Sample Preparation

Fresh whole blood was collected by venipuncture in K3-EDTA-containing tubes; after collection, tubes were promptly covered in aluminum foil to ensure light protection and stored at 4 °C until centrifugation. Tubes were centrifuged at 3500× *g* for 15 min at 4 °C and plasma was collected. Plasma samples were either processed or stored at −40 °C until further analysis. 

Samples were extracted by acidic protein precipitation. Aliquots of 100 µL of plasma samples were dispensed in 1.5 mL safe-lock tubes; 300 µL of a 10% TFA solution spiked with the IS L-DOPA-D3 at the concentration of 200 µg/L were added to the tubes. Samples were then vortexed for 10 s and centrifuged at 13,000 rpm for 10 min at 4 °C. The supernatant was collected and transferred to a clean 1.5 mL safe-lock tube, which underwent a further step of centrifugation at 13,000 rpm for 5 min at 4 °C. Finally, 250 µL of clean supernatant was collected and transferred to clean glass autosampler vials; 5 µL of sample was injected into the instrument.

### 4.4. Instrument Setup and Parameters

LC-MS/MS analysis was carried out on a Waters Xevo TQ-XS triple quadrupole mass spectrometer coupled to a Waters Acquity UPLC I-Class System (Waters Corporation, Sesto San Giovanni, Italy). Chromatographic separation was performed on a Kinetex PFP column (50 × 2.1 mm; 2.6 µm particle size) (Phenomenex, Torrance, CA, United States) with two mobile phases: mobile phase A consisted of 0.1% formic acid in water while mobile phase B consisted of 0.1% formic acid in acetonitrile. Elution was performed in gradient elution mode: at a flow rate of 0.4 mL/min, the gradient was stepped from 0% B (100% A) to 30% B (70% A) in 2 min. Then, the column was washed for 1 minute at 70% B (30% A) and restored to the initial condition of 0% B (100% A) for column equilibration. Total run time was 4.5 minutes; column oven was set at 22 °C.

All compounds were detected in multiple reaction monitoring (MRM) mode as described in Table 3. The detecting conditions for each compound were obtained by directly infusing standard solutions in 50% MeOH at 250 µg/L.

MS analysis was performed using a triple quadrupole mass spectrometer equipped with an electrospray ionization (ESI) source. Method parameters were the following: source temperature +150 °C, capillary voltage +1.5 kV, desolvation temperature +500 °C, cone gas flow rate 150 L/h, collision gas flow rate 0.15 mL/min, and desolvation gas flow rate 1000 L/h. Dwell time was set to 0.008 s. Waters MassLynx (V 4.2) and TargetLynx software were used to acquire and process data (Waters Corporation, Sesto San Giovanni, Italy).

### 4.5. Method Validation

Method validation was performed according to the most recent International Council for Harmonization (ICH) guidelines for bioanalytical method validation “ICH Guideline M10 on Bioanalytical Method Validation and Study Sample Analysis” released by EMA Committee for Medicinal Products for Human Use on 25th July 2022 [34]. Required validation steps are selectivity and specificity, calibration curve linearity, carry-over, dilution integrity, matrix effect, accuracy and precision, reinjection reproducibility, and stability.

#### 4.5.1. Selectivity and Specificity

Method selectivity and specificity were assessed by analyzing six different lots of blank matrices and assessing the absence of significant response accountable to interfering components at analytes and internal standard retention times. Response should not be greater than 20% of analyte response at LLOQ and 5% of IS response for each matrix.

#### 4.5.2. Linearity

Calibration curves were built by plotting analyte/IS peak areas ratio against corresponding analyte nominal concentrations and fitted by using a 1/x weighted linear regression model.

To assess LLOQ, spiked plasma samples at the concentrations of 100, 75, 50, 25, 15, 10, 5, and 1 µg/L were prepared and analyzed in triplicate. LLOQ was defined as the lowest concentration level at which accuracy and precision were within ±20%.

To assess ULOQ, spiked plasma samples at the concentrations of 3000, 4000, 6000, 8000, and 10,000 µg/L were prepared and analyzed in triplicate. ULOQ was defined as the highest concentration level at which calibration curve maintained its linearity by precision and accuracy being within ±15%.

#### 4.5.3. Carry Over

Carryover was assessed by analyzing six blank plasma samples after the injection of a calibration standard at the ULOQ. Response should not be greater than 20% of analyte response at LLOQ and 5% of IS response for each blank sample.

#### 4.5.4. Dilution Integrity

Dilution integrity was performed to assess the viability of the dilution procedure. A high-concentrated plasma sample (15,000 µg/L for L-DOPA and LDME, 10,000 for carbidopa) was serially diluted with blank plasma to obtain 1:2 and 1:10 dilutions falling within the calibration range. Diluted samples were analyzed in triplicate. The mean accuracy and precision of diluted samples should be within ±15%.

#### 4.5.5. Recovery and Matrix Effect

RE and ME were assessed for each analyte at three concentration levels (LQC, MQC, and HQC) by analyzing six different lots of matrices in triplicate. RE and ME were calculated according to Matuszewski [35]; IS normalization was applied as described in De Nicolò et al. [36]; thus, ISn-ME and ISn-RE values are shown. 

#### 4.5.6. Accuracy and Precision

Intra-day and inter-day accuracy and precision were assessed at four concentration levels (LLOQ, LQC, MQC, and HQC) with a 5 × 5 scheme, consisting of five replicates for each concentration level in five analytical runs over five nonconsecutive days.

Accuracy is expressed as relative error, or bias%, which is calculated as the percentual difference between measured and nominal concentrations, while precision is expressed as CV%. Mean accuracy and precision values within ±15% for LQC, MQC, HQC, and within ±20% for LLOQ were considered acceptable.

#### 4.5.7. Reinjection Reproducibility

Reinjection reproducibility was evaluated to establish the viability of processed samples. One run from accuracy and precision experiments was reinjected after storage in the autosampler compartment (10 °C) for 24 h and accuracy and precision of reinjected QCs were assessed.

#### 4.5.8. Stability

Sample stability was measured at two concentration levels (LQC and HQC) in triplicate. Long-term stability was evaluated at −40 °C for 2 weeks, 1 and 2 months; short-term stability was evaluated at room temperature (rt) under light exposure and with light protection and at 4 °C (light protection) for 1, 2, 3, 4, and 7 days. Autosampler stability was also assessed by storing extracts in the autosampler compartment at 10 °C for 1, 2, 3, 4, and 7 days.

The mean concentration values obtained from each measurement were compared to the concentrations measured at T0 before storage. Stability data are expressed as percentual difference from T0.

#### 4.5.9. Statistical and Graphical Analysis

MedCalc^®^ (version 20.215, MedCalc Software bv, Ostend, Belgium) was used for PK parameters determination.

## Figures and Tables

**Table 1:** (**a**) Inter-day precision and accuracy of the quantification method in human plasma. QCs = quality control samples; LLOQ = lower limit of quantification (15 µg/L); LQC = low QC (300 µg/L); MQC = medium QC (900 µg/L); HQC = high QC (1800 µg/L); CV = coefficient of variation. (**b**) Intra-day precision and accuracy of the quantification method in human plasma. QCs = quality control samples; LLOQ = lower limit of quantification (15 µg/L); LQC = low QC (300 µg/L); MQC = medium QC (900 µg/L); HQC = high QC (1800 µg/L); CV = coefficient of variation.

(a)																
INTER-DAY																
		L-DOPA					LDME					Carbidopa				
QCs		D1	D2	D3	D4	D5	D1	D2	D3	D4	D5	D1	D2	D3	D4	D5
LLOQ	Bias%	0.6	4.0	−7.9	−5.5	−0.5	6.7	−4.9	11.8	10.7	−17.4	−0.1	5.9	10.2	8.1	−10.1
	CV%	3.4	8.5	2.6	4.0	5.5	8.7	4.1	5.2	5.0	2.9	4.4	6.3	8.2	5.5	9.5
LQC	Bias%	−0.3	11.4	3.1	2.9	−4.1	10.4	−9.4	2.6	7.7	−9.5	2.5	−5.7	1.5	4.3	−9.6
	CV%	3.1	2.9	2.1	2.5	2.7	3.1	4.9	6.3	6.7	2.8	4.1	4.3	5.6	8.1	5.9
MQC	Bias%	−1.0	0.6	−4.3	−1.3	−7.3	8.6	−7.3	−3.1	5.9	−12.7	8.1	−0.1	4.7	9.2	−6.3
	CV%	6.0	4.5	2.3	2.7	1.6	2.3	7.1	5.6	4.0	2.1	2.3	3.6	4.9	4.5	2.1
HQC	Bias%	−2.2	−4.0	−4.7	−5.4	−3.1	−0.1	−7.8	5.3	4.3	−9.4	2.9	−7.3	−0.1	2.5	−3.5
	CV%	2.4	3.4	2.0	2.4	5.0	7.2	4.6	4.5	6.1	3.9	5.0	2.5	4.1	7.3	4.2
**(b)**																
**INTRA-DAY**																
**QCs**		**L-DOPA**					**LDME**					**Carbidopa**				
LLOQ	Bias%	0.7					4.5					1.4				
	CV%	7.6					11.0					7.9				
LQC	Bias%	−0.3					1.9					−2.5				
	CV%	8.6					9.7					6.1				
MQC	Bias%	−2.8					1.4					1.8				
	CV%	3.0					10.5					5.5				
HQC	Bias%	−3.1					1.7					−1.2				
	CV%	2.2					10.8					3.9				

**Table 2:** PK parameters after administration of two tablets of an LDME/carbidopa (100/25 mg) formulation compared to plasma concentrations after the administration of 500 mg of L-DOPA from commercially available *Mucuna pruriens* extracts.

Formulation	Dose (mg)	Cmax (µg/L)	Tmax (min)	Cmin (µg/L)	t_1/2_ (min)	AUC (µg × h/mL)
LDME/Carbidopa	2 × 100/25	2795	15	323	40	2.687
*Mucuna pruriens* extract	500	3597	30	1293	141	7.043
		3482	60			

**Table 3:** MS method transitions (in bold quantifier ion, in italic qualifier ion), parameters, and retention times.

Compound	Precursor Ion (*m/z*)	Product Ion (*m/z*)	Cone (V)	Collision (V)	Retention Time (min)
L-DOPA	198.09	**152.13**	15	15	0.86
		*134.94*	20	16	
LDME	212.50	**152.13**	20	14	1.59
		*134.92*	20	18	
Carbidopa	227.07	**181.11**	20	15	1.44
		*123.08*	20	27	
L-DOPA-D3	201.05	**154.39**	20	16	0.85

## Data Availability

The data that support the findings of this study are available from the corresponding author upon reasonable request.

## Supplementary Materials

The following supporting information can be downloaded at: https://www.mdpi.com/article/10.3390/molecules28114264/s1, Figure S1: L-DOPA, LDME, and carbidopa stability; Table S1: Matrix effect (ME) and recovery (RE) data.
