# Supplementary material for: Development and Validation of a New LC-MS/MS Bioanalytical Method for the Simultaneous Determination of Levodopa, Levodopa Methyl Ester, and Carbidopa in Human Plasma Samples
Source: Molecules. 2023 May 23;28(11):4264. doi: 10.3390/molecules28114264 (PMC10254897; doi:10.3390/molecules28114264)

Table S1. Summary of matrix effect (ME) and recovery (RE) data and internal standard normalized (ISn) values (n=6). L-DOPA (Levodopa); LDME (Levodopa-methyl-ester); L-DOPA-d3 (Levodopa-d3); LQC = low QC (300 μg/L); MQC = medium QC (900 μg/L); HQC = high QC (1800 μg/L).

MATRIX EFFECT AND RECOVERY													
		L-DOPA			LDME			Carbidopa			L-DOPA-d3		
		LQC	MQC	HQC	LQC	MQC	HQC	LQC	MQC	HQC	LQC	MQC	HQC
ME%	Mean	102	105	102	97	98	97	107	106	98	100	98	95
	SD	2.9	2.6	3.8	6.6	4.1	4.1	4.7	3.4	1.8	4.2	0.9	3.0
ISn-ME%	Mean	102	107	108	97	100	103	107	109	104	-	-	-
	SD	6.8	2.4	4.3	8.0	3.9	5.6	7.0	3.1	4.4	-	-	-
RE%	Mean	113	106	104	77	77	77	95	88	88	101	100	93
	SD	2.8	6.7	4.6	7.0	6.5	6.3	6.2	5.9	2.3	4.5	3.6	4.9
ISn-RE%	Mean	112	106	112	76	77	83	94	89	95	-	-	-
	SD	7.3	6.9	8.4	5.0	5.9	4.2	6.5	3.3	4.4	-	-	-

Figure S1. L-DOPA, LDME and Carbidopa (C-DOPA) short-term and medium-term stability in plasma samples and extracts at different storage times and conditions (n=3). Stability is expressed as percentage difference from T0 (% of degradation). Reference lines show acceptability range within ± 15%.

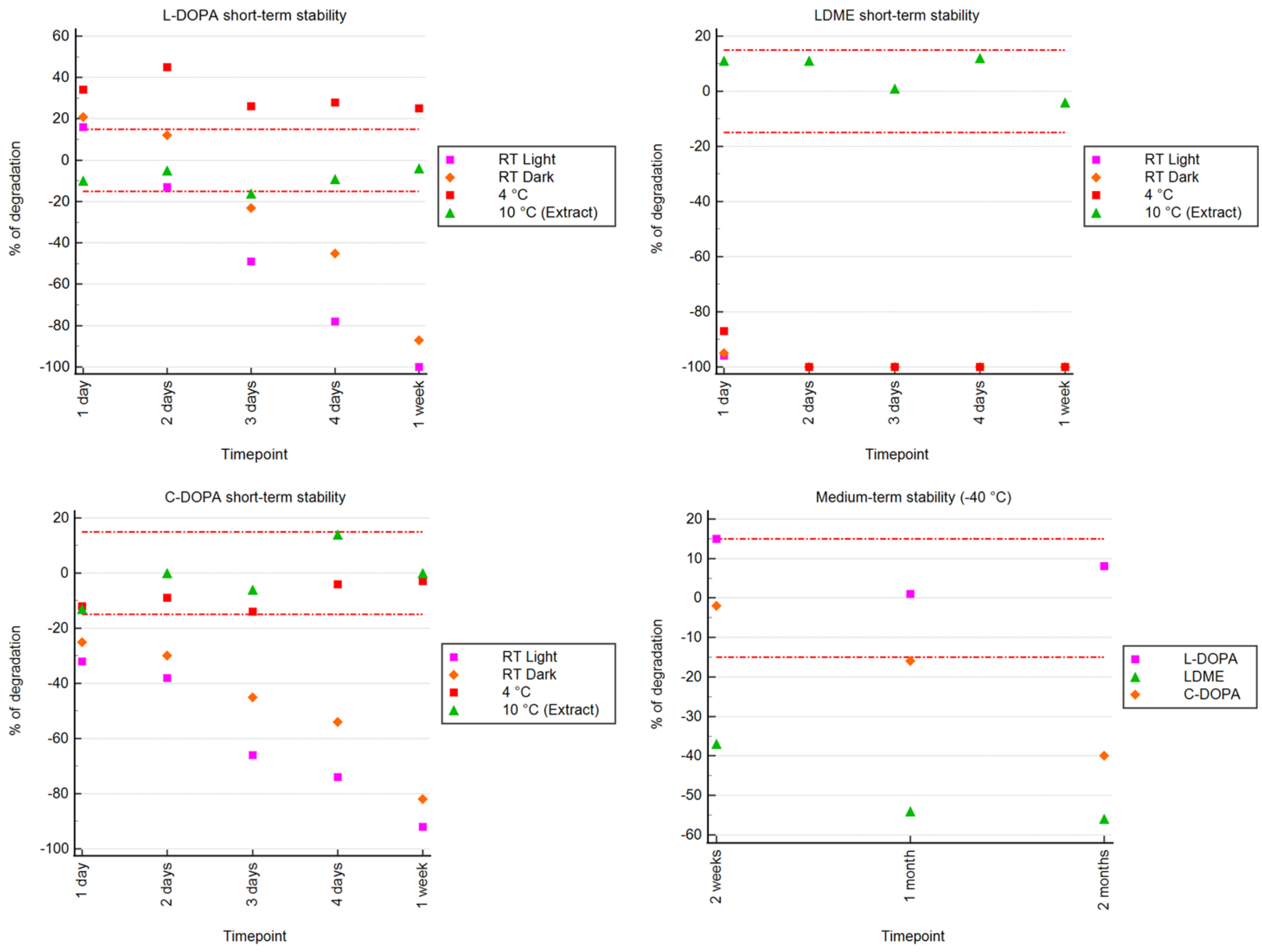

Supplement: Supplementary file 1 [file molecules-28-04264-s001.zip › molecules-2329631-supplementary.pdf]
